# Molecular diversity and phylogenetic characterization of the *invA* gene in non-typhoidal *Salmonella* from domestic animals and wildlife in Zambia

**DOI:** 10.14202/vetworld.2026.1824-1837

**Published:** 2026-05-03

**Authors:** Charles Miyanda Mubita, John Bwalya Muma, Joseph Ndebe, Edgar Simulundu, Bernard Mudenda Hang’ombe

**Affiliations:** 1 Department of Medicine and Clinical Sciences, School of Medicine, Eden University, Lusaka 10101, Zambia; 2 Department of Disease Control, School of Veterinary Medicine, University of Zambia, Lusaka 10101, Zambia; 3 Macha Research Trust, Choma 20100, Zambia; 4 Division of International Research Promotion, International Institute for Zoonosis Control, Hokkaido University, Sapporo 001-0020, Japan

**Keywords:** domestic animals, *invA* gene, molecular diversity, non-typhoidal *Salmonella*, phylogenetic analysis, wildlife interface, Zambia, zoonotic transmission

## Abstract

**Background and Aim:**

Non-typhoidal *Salmonella* remains a significant zoonotic pathogen with substantial implications for animal and public health, particularly in regions where domestic animals and wildlife share ecological interfaces. The invasion protein A (*invA*) gene is widely used as a molecular marker for detecting *Salmonella*; however, its sequence variability across host species in sub-Saharan Africa remains poorly understood. This study aimed to characterize nucleotide diversity and phylogenetic relationships of the *invA* gene among *Salmonella* isolates obtained from domestic animals and free-ranging wildlife in selected regions of Zambia.

**Materials and Methods:**

A total of 12 *invA* gene amplicons derived from previously confirmed *Salmonella* isolates from domestic animals (n = 10) and wildlife (n = 2) were selected for sequencing. Polymerase chain reaction products were purified and sequenced using BigDye terminator chemistry on a 3500 Genetic Analyzer. Sequence assembly and editing were performed using GENETYX software. Multiple sequence alignment was performed with ClustalW, and phylogenetic relationships were inferred using maximum-likelihood with the Tamura–Nei model in MEGA7. Comparative analysis included eight reference sequences retrieved from GenBank.

**Results:**

The *invA* gene sequences demonstrated high nucleotide similarity (98.0%–100.0%) among isolates from domestic animals and wildlife, while showing broader variability (74.0%–100.0%) when compared with global reference strains. Six distinct sequence types were identified, with the majority originating from chicken isolates. Nucleotide substitutions were predominantly observed between positions 40 bp and 250 bp, indicating a potential hotspot for genetic variation. Some substitutions resulted in amino acid changes, suggesting possible structural and functional implications for the invasion protein. Phylogenetic analysis revealed that all Zambian isolates clustered within a single major lineage (Clade A), indicating close genetic relatedness across host species.

**Conclusion:**

This study provides the first evidence of *invA* gene sequence polymorphism among Salmonella isolates from domestic animals and wildlife in Zambia. The findings highlight the dynamic evolution of Salmonella at the wildlife–livestock interface and underscore the importance of molecular surveillance in understanding zoonotic transmission. The identified sequence variations may have implications for diagnostic accuracy and the development of region-specific detection tools, reinforcing the need for continuous genomic monitoring under a One Health framework.

## INTRODUCTION

Non-typhoidal *Salmonella* remains a major zoonotic pathogen affecting humans, livestock, and wildlife [1–4], particularly in regions where ecological overlap and shared resources facilitate cross-species transmission [5, 6]. It is estimated that *Salmonella* causes 93.8 million cases of gastroenteritis and 155,000 deaths per year worldwide [7]. The presumptive identification of *Salmonella* isolates usually depends on phenotypic characteristics expressed on selective media and biochemical tests such as triple sugar iron agar and urease testing [8]. These conventional microbiological methods are labor-intensive and time-consuming to produce the desired results [9].

To overcome these challenges, a variety of rapid and robust molecular techniques have been developed, among which the polymerase chain reaction (PCR) has been widely applied [10]. PCR is an effective tool for the identification of *Salmonella* by amplification of the invasion protein A (*invA*) gene [11–14]. The *invA* gene is located on *Salmonella* pathogenicity island 1 and encodes a type III secretion system that produces proteins responsible for the invasion of epithelial cells of the host [15]. The *invA* gene is currently considered an international standard parameter because it contains a unique nucleotide sequence that is targeted to identify bacteria of the *Salmonella* genus [12, 14, 16, 17].

Using PCR methods, various prevalence rates of *Salmonella* have been reported across animal reservoirs and geographic regions, including India [11], the Philippines [18], and Turkey [19]. Similarly, several studies have used the *invA* gene to determine the prevalence of *Salmonella* in foods of animal origin in Zambia [20, 21]. A large number of domestic animals and wildlife are plausible reservoirs of zoonotic infection [22, 23]. A study conducted in the Giza Governorate of Egypt examined relationships among *invA* gene nucleotide sequences from diverse sources using phylogenetic analysis, incorporating isolates from human stool, egg samples, and poultry [13, 24].

Despite the widespread use of the *invA* gene as a reliable molecular marker for the detection of *Salmonella* enterica, existing studies in Zambia have largely been limited to presence–absence detection and prevalence estimation using polymerase chain reaction-based approaches [20, 21]. These investigations, although valuable for epidemiological surveillance, do not provide insights into sequence-level variation, genetic polymorphism, or evolutionary dynamics of the *invA* gene across different host species. Furthermore, there is a paucity of data examining whether *invA* gene sequences exhibit host-associated adaptations or divergence between isolates originating from domestic animals and free-ranging wildlife within shared ecological systems. This represents a critical gap, particularly in regions such as Zambia, where wildlife–livestock interfaces facilitate frequent cross-species interactions and potential bidirectional transmission of zoonotic pathogens [5, 6].

In addition, global studies have demonstrated that virulence-associated genes, including *invA*, may harbor nucleotide substitutions and mutation hotspots that can influence protein structure, pathogenicity, and the sensitivity of molecular diagnostic assays. However, such sequence-based analyses remain largely unexplored in sub-Saharan Africa, and no study to date has systematically characterized *invA* gene polymorphism and phylogenetic relationships among *S. enterica* isolates from both domestic and wildlife reservoirs within a unified analytical framework. The absence of region-specific genomic data further limits the ability to design optimized diagnostic tools and to understand the evolutionary ecology of *Salmonella* in diverse host populations.

Therefore, addressing this knowledge gap is essential for moving beyond conventional detection toward a deeper understanding of the genetic diversity, transmission dynamics, and adaptive evolution of *S. enterica*. Such information is crucial for strengthening molecular surveillance systems, improving diagnostic accuracy, and informing integrated One Health strategies to control zoonotic salmonellosis in Zambia and similar ecological settings.

Therefore, this study aimed to (i) characterize nucleotide and amino acid polymorphisms within the *invA* gene, (ii) identify distinct sequence types and potential mutation hotspots, (iii) assess phylogenetic clustering and genetic relatedness among isolates from diverse host species, and (iv) evaluate the implications of observed genetic variability for zoonotic transmission at the wildlife–livestock interface. By integrating molecular sequencing with phylogenetic inference within a One Health framework, this study further sought to generate baseline genomic data to inform region-specific diagnostic tool development, enhance surveillance strategies, and improve understanding of the evolutionary ecology of *S. enterica* in sub-Saharan Africa.

## MATERIALS AND METHODS

### Ethical approval

This study used *S. enterica* isolates that had been recovered previously from domestic animals and free-ranging wildlife sampled in selected areas of Zambia. According to the study context described in the manuscript, formal ethical and biosafety approval was not required at the time of sample collection for handling bacterial isolates obtained from domestic animals. Wildlife sampling was conducted under research quotas and permissions previously authorized by the Zambia Wildlife Authority. No experimental infection, invasive animal handling for the purpose of this sequencing work, or additional live-animal intervention was performed as part of the present study. Downstream laboratory procedures were conducted on previously recovered isolates, and all microbiological and molecular analyses were performed in accordance with standard institutional biosafety practices. In particular, biosafety level 2 precautions were followed during isolate handling, deoxyribonucleic acid extraction, PCR setup, amplicon processing, and sequencing workflows, including the use of sterile workspaces, dedicated reagents, and separation of pre- and post-PCR areas to minimize contamination and ensure laboratory safety.

### Study period and location

The study was conducted from January 2013 to December 2019. Cattle samples were collected from seven districts in Southern Province: Mazabuka (n = 118), Monze (n = 126), Pemba (n = 126), Choma (n = 125), Kalomo (n = 50), Zimba (n = 28), and Kazungula (n = 59). All faecal samples from horses were collected in Lusaka Province. Wildlife samples were obtained from Kafue National Park (KNP), Lochinvar National Park (LNP), and Central National Park (CNP).

### Study design and sample selection

The original samples were collected from free-ranging wildlife and domestic animals across selected areas of Zambia from June 2013 to December 2014 [25]. Sampling and specimen collection were conducted opportunistically, based on the availability of animal species considered potential reservoirs of the pathogen. A total of 1,248 samples from domestic animals (1,008) and wildlife (240) were collected and analyzed for *Salmonella* [25]. Of the domestic animal samples, 659 (65.4%) were fecal samples from apparently healthy animals (cattle = 632; horses = 27), while 349 (34.6%) were samples from diseased animals submitted for routine microbiological diagnosis. The distribution of samples across the various wildlife sources and geographic locations, including Kafue National Park (KNP), Lusaka Central Park, and Lochinvar National Park (LNP), is presented in Table 1. All samples were characterized through conventional identification methods [26].

**Table 1:** Distribution of fecal samples of wildlife (n = 240) from KNP, LNP and LCP.

Host	Scientific name	KNP	LNP	LCP
Baboon	*Papio anubis*	0	21	0
Lion	*Panthera leo*	14	0	0
Porcupine	*Erethizon dorsatum*	1	0	0
Puku antelope	*Kobus vardonii*	59	0	0
Water buck	*Kobus ellipsiprymnus*	16	0	0
Cheetah	*Acinonyx jubatus*	1	0	0
Impala	*Aepyceros melampus*	42	0	0
Warthog	*Phacochoerus africanus*	3	0	0
Wild dogs	*Lycaon pictus*	10	0	0
Leopard	*Panthera pardus*	6	0	0
Hartebeest	*Alcelaphus buselaphus*	2	0	0
Hippopotamus	*Hippopotamus amphibius*	9	0	0
Lechwe	*Kobus leche kafuensis*	29	0	0
Elephant	*Loxodonta africana*	10	0	0
Roan antelope	*Hippotragus equinus*	2	0	0
Buffalo	*Bubalus bubalis*	6	0	0
Sable antelope	*Hippotragus niger*	0	0	9
Total		210	21	9

KNP = Kafue National Park LNP = Lochnivar National Park LCP = Lusaka Central Park

### Molecular characterization of *Salmonella* strains

*Salmonella* strains isolated from the range of domestic animals and free-ranging wildlife were examined for the presence of the *invA* gene [25].

Deoxyribonucleic acid extraction and PCR amplification of *invA* gene: Genomic DNA was isolated from each *Salmonella* isolate using a DNeasy Blood and Tissue Kit (Qiagen, Hilden, Germany) following the manufacturer’s protocol. A single colony was suspended in distilled water (200 μL), lysed with DNAzol (200 μL), and incubated at 25°C for 5 days to ensure complete cell disruption. DNAzol (800 μL) was added, and the mixture was further incubated at 25°C for 30 min. The lysate was clarified by centrifugation at 10,000 × *g* for 10 min at 4°C, and DNA (750 μL) was precipitated with absolute ethanol (375 μL), mixed thoroughly by vortex. The mixture was centrifuged at 12,000 × *g* for 10 min at 4°C. After sequential washes with 75% ethanol, the pellet in the microcentrifuge tube was left to dry completely at 25°C for 10 min, then resuspended in 8 mM sodium hydroxide (200 μL). The suspension was incubated for half a day at 4°C, then centrifuged at 12,000 × *g* for 10 min at 4°C. The solution (200 μL) was neutralized with 2 μL of 4-(2-hydroxyethyl)-1-piperazineethanesulfonic acid (Sigma Adrich, Steinheim, Gemerny), and the recovered genomic DNA was stored at −30°C until analysis. To minimize contamination during DNA extraction and downstream molecular procedures, all steps were performed on a sterile workstation using dedicated reagents and separate pre- and post-PCR areas.

Selection of DNA primers and detection of *invA* gene: The forward and reverse primers (Inqaba Biotechnical Industries (Pty) Ltd, Pretoria, South Africa) at specific molecular sizes were used to detect the presence of a stable genetic region of the *invA* gene of *Salmonella*, following the protocol described by Rahn *et al*. [27]. The working concentration of each primer was 10.0 μM and the oligonucleotide sequences are shown in Table 2.

**Table 2:** Uniplex set of oligonucleotides (primers) used for polymerase chain reaction amplicons.

Primer	Target oligonucleotide sequence	Length	Reference
*invA* F	5’-GTGAAATTATCGCCACGTTCGGGCAA-3’	284 bp	[27]
*invA* R	5’–TCATCGCACCGTCAAAGGGAACC–3’		

*invA* = invasion protein A gene PCR = Polymerase Chain Reaction bp = base pairs

PCR amplification of the *invA* gene was carried out following the primer set originally described by Rahn *et al*. [27]. Each 10 μL reaction consisted of nuclease-free water (6.75 μL), 10× buffer (1.0 μL; Applied Biosystems, Warrington, UK), forward (0.2 μL) and reverse (0.2 μL) *invA* primers, deoxynucleotide triphosphates (0.8 μL; Takara Bio Inc., Shiga, Japan), Ex Taq polymerase (0.05 μL; Takara Bio Inc.), and 1 μL of DNA template. Reactions were assembled on ice and immediately transferred to a thermocycler (Applied Biosystems AB-Veriti, Foster City, CA, USA) preheated to 95°C. The cycling protocol included an initial denaturation at 95°C for 1 min, followed by 35 cycles of 95°C for 30 s, 55°C for 30 s, and 72°C for 30 s, with a final extension at 72°C for 5 min before holding at 4°C.

Thirty-four *Salmonella* strains previously isolated from various domestic animals and free-ranging wildlife samples were molecularly confirmed by detection of the *invA* gene [25]. All *Salmonella* isolates were referred to the Deltamune (Pty) Laboratory, Pretoria, South Africa, for serological confirmation and serotyping [25]. Twelve distinct serotypes were identified among the 34 strains of *Salmonella* isolated from domestic animals and free-ranging wildlife (*p* = 0.002, 95% confidence interval). The distribution of *Salmonella* serotypes by animal source is shown in Table 3.

**Table 3:** Distribution of *Salmonella* *enterica* subsp. Enterica serotypes isolated from domestic animals and wildlife.

Serotype	Cattle	Horse	Chicken	Dog	Leopard	Sable	Impala	Total
*S.* Heidelberg	2	-	-	-	-	-	-	2
*S.* Enteritidis	-	1	15	-	-	-	-	16
*S.* Garoli	-	-	-	-	1	-	-	1
*S.* Pomona	-	-	-	-	-	1	-	1
*S.* Roan	-	-	-	-	-	-	1	1
*S.* Ruanda	-	-	2	-	-	-	-	2
*Salmonella spp.*	-	-	2	-	-	-	-	2
*S.* Hadar	-	-	1	-	-	-	-	1
*S.* Stockholm	-	-	2	-	-	-	-	2
*S.* Chardan	-	-	2	-	-	-	-	2
*S.* Sendai	-	-	1	-	-	-	-	1
*S.* Mbandaka	-	-	1	2	-	-	-	3
Total	2	1	26	2	1	1	1	34

Selection and sequencing of polymerase chain reaction products: In the present study, 12 PCR *invA* gene PCR amplicon products derived from *Salmonella* isolates obtained from domestic animals (n = 10) and from two free-ranging wildlife sources – sable antelope and leopard were purposively selected from strains of a previous study [25] and were targeted for PCR product sequencing (Table 4). Among the strains from domestic animals, 8 (80%) were from chickens, while 1 isolate each (10%) was obtained from cattle and a horse. All chicken strains were derived from clinically symptomatic hosts.

**Table 4:** Distribution of *Salmonella* *enterica* subsp. *enterica* serotypes sequenced from domestic animals and free-ranging wildlife (n = 12).

Source	Isolate ID	Accession No.	Species	Serotype	No. of strains
Cattle	670 (ZM.CTL)	PP921861	enterica	*S.* Heidelberg	1
Horse	B10 (ZM_HSE)	PP921862	enterica	*S.* Enteritidis	1
Chicken	WF1-3 (ZM_CHK)	PP921853	enterica	*S.* Enteritidis	1
Chicken	P12 (ZM_CHK)	PP921860	enterica	*S.* Ruanda	1
Chicken	P13e (ZM_CHK)	PP921854	enterica	*S.* Ruanda	1
Chicken	P19 (ZM_CHK)	PP921855	enterica	*Salmonella* spp.	1
Chicken	B1 (ZM_CHK)	PP921856	enterica	*S.* Hadar	1
Chicken	B5 (ZM_CHK)	PP921857	enterica	*S.* Stockholm	1
Chicken	B7 (ZM_CHK)	PP921858	enterica	*S.* Chardan	1
Chicken	P21 (ZM_CHK)	PP921859	enterica	*S.* Sendai	1
Subtotal					10
Wildlife					
Sable antelope	SI-I (ZM_SBL)	PP921852	enterica	*S.* Pomona	1
Leopard	3 (ZM_LPD)	PP921851	enterica	*S.* Garoli	1
Subtotal					2
Grand Total					12

ZM = Zambia, CTL = Cattle, HSE = Horse, CHK = Chicken, SBL = Sable antelope, LPD = Leopard, S. = *Salmonella* spp. = species (plural) n = number

**Purification of sequencing reactions:** Sequencing products were cleaned using an ethanol/ ethylenediamine-tetraacetic acid/sodium acetate precipitation protocol recommended by the manufacturer (Applied Biosystems, Foster City, CA, USA). Each reaction was mixed with 125 mM ethylenediaminetetraacetic acid (2.0 μL) and 3 M sodium acetate (2.0 μL), followed by the addition of absolute ethanol (90 μL) to facilitate DNA precipitation. After a brief incubation in the dark at 25°C for 10 min, samples were pelleted by high-speed centrifugation (20,000 × g for 20 min), washed twice with 200 μL of 70% ethanol, and air dried. The DNA was then dissolved in formamide, denatured at 95°C for 3 min, and subsequently loaded onto a 3500 Genetic Analyzer for capillary electrophoresis.

**Sequence assembly and editing:** Chromatograms of the nucleotide sequences obtained from *Salmonella* strains from domestic animals and wildlife (n = 12) were inspected, assembled, and edited using GENETYX version 12 (GENETYX Corporation, Tokyo, Japan). Final consensus sequences were used for downstream analyses. Consensus sequences were compared with reference sequences available in GenBank to identify nucleotide differences using the ClustalW multiple-alignment algorithm (https://www.genome.jp/tools-bin/clustalw accessed on 20 February 2025). Specific nucleotide variations were verified by comparing the raw chromatogram peaks and the consensus sequences generated from each primer. Unreliable regions at the 5′ and 3′ ends of the consensus sequences were trimmed based on the quality of the chromatogram peaks for each primer. Only nucleotide positions that showed consistent, high-quality peaks from both forward and reverse reads were retained. Bases with poor or ambiguous peaks, particularly at the terminal regions of the sequences, were removed during trimming.

### Phylogenetic analysis

The nucleotide sequences obtained from *Salmonella* strains from domestic animals (n = 10) and wildlife (n = 2) were assembled and edited using the GENETYX version 12 software. Multiple alignments between nucleotide and amino acid sequences of *Salmonella* were performed based on the ClustalW method (https://www.genome. jp/tools-bin/clustalw accessed on 20 February 2025). Reference sequences of *Salmonella* were also downloaded from the National Center for Biotechnology Information GenBank (n = 8) and compared with those from domestic animals and free-ranging wildlife. Further reference sequences that were shorter than the study strains were removed from the analysis.

The evolutionary history of nucleotides and amino acids in the strains was analyzed using maximum-likelihood methods. The model was selected based on the Bayesian information criterion. Based on this criterion, the Tamura–Nei evolutionary model had the lowest Bayesian information criterion score and was thus selected as the best model for the trees. The trees were constructed using MEGA7 version software [28]. The reliability of the branching order of the trees was determined using 1000 bootstrap replicates. Twenty *invA* gene sequences from local strains (n = 12) and international reference strains (n = 8) were included in the phylogenetic tree (Figure 3).

The nucleotide sequences of the *invA* gene of *Salmonella* isolates from domestic animals and free-ranging wildlife were deposited in GenBank under accession numbers PP921856–PP921862.

At the time of sample collection, ethical and biosafety approval were not required for handling *Salmonella* isolates from domestic animals. Wildlife samples were collected according to research quotas previously authorized by the Zambia Wildlife Authority [25]. Nevertheless, all biosafety level 2 procedures were strictly followed during the downstream handling and processing of the samples.

### Data availability

Sequence alignments were saved in ClustalW (.aln) format, and the resulting phylogenetic trees were stored as MEGA tree (.mts) files for future accessibility.

## RESULTS

### Multiple sequence alignment of *invA* gene fragment

**Figure 1:**
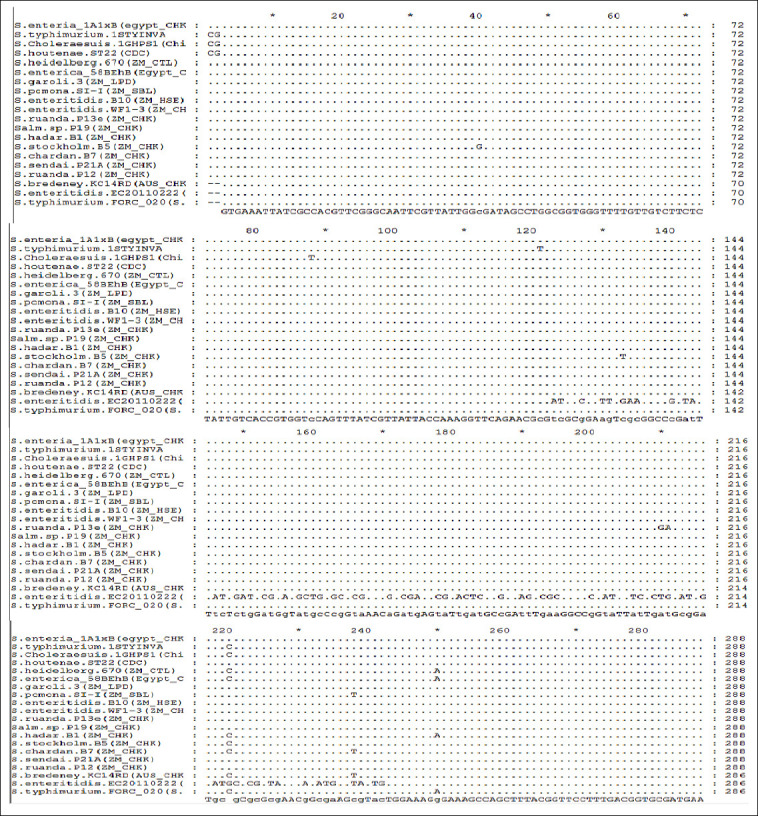
Multiple alignments of nucleotide sequences of protein invasion A (*invA*-284 bp) gene of local *Salmonella enterica* serotype strains isolated from domestic animals and wildlife compared with sequences of reference *S. enterica* strains retrieved from GenBank.

Diversity in *invA* sequences from both the intra- and inter-serovar *Salmonella enterica* subsp. enterica serotypes were observed when 12 *S. enterica* strains that were sequenced in this study were aligned with eight reference sequences of the *invA* gene available from the National Center for Biotechnology Information (NCBI) (Figure 1 and Table 5).

**Table 5:** Distribution of *invA* nucleotide sequence types among the *Salmonella* strains (n = 12).

Type	Serotype	Accession No.	Host	40	88	121	133	210	211	220	238	250
				C	C	C	C	A	T	T	C	G
I	*S.* Garoli.3	PP921851	Leopard	.	.	.	.	.	.	.	.	.
I	*S.* Enteritidis.B10	PP921862	Horse	.	.	.	.	.	.	.	.	.
I	*S.* Enteritidis.WF1-3	PP921853	Chicken	.	.	.	.	.	.	.	.	.
I	*Salmonella* spp. P19	PP921855	Chicken	.	.	.	.	.	.	.	.	.
I	*S.* Sendai.P21	PP921859	Chicken	.	.	.	.	.	.	.	.	.
I	*S.* Ruanda.P12	PP921860	Chicken	.	.	.	.	.	.	.	.	.
II	*S.* Heidelberg.670	PP921861	Cattle	.	.	.	.	.	.	C	.	A
II	*S.* Hadar.B1	PP921856	Chicken	.	.	.	.	.	.	C	.	A
III	*S.* Pomona.SI-I	PP921852	Sable	.	.	.	.	.	.	.	T	.
IV	*S.* Ruanda.P13e	PP921854	Chicken	.	.	.	.	G	A	.	.	.
V	*S*. Stockholm.B5	PP921857	Chicken	G	.	.	T	.	.	C	.	.
VI	*S.* Chardan.B7	PP921858	Chicken	.	.	.	.	.	.	C	T	.

*invA* = invasion protein A gene, S. = *Salmonella,* spp. = species (plural), n = number, . = no mutation (identical to consensus), C, A, T, G = nucleotide bases (Cytosine, Adenine, Thymine, Guanine).

The comparison revealed substitution differences mainly in the region between 40 bp and 250 bp positions of the nucleotide sequence of the *invA*-284 bp gene sequenced in this study. A substitution was observed where one of these bases, adenine, cytosine, guanine, and thymine, was replaced by another. Further analysis of the *invA* nucleotide sequence differences revealed six distinct sequence types among the 12 strains from domestic animals and free-ranging wildlife strains. Of these, sequence type I was the most predominant, with 50% (6/12) of the strains being 100% identical. These strains were indistinguishable from the reference international strains with accession numbers (*S. enterica* subsp. enterica KJ718884.1) and *S. enterica* subsp. enterica serovar Houtenae ST22 (DQ644627.1) from Egypt and the Centers for Disease Control and Prevention, respectively. Sequence variation was classified according to the position and type of single-nucleotide polymorphisms identified between positions 40 bp and 250 bp within the 280 bp *invA* gene fragment of S. enterica.

On the other hand, 50% (6/12) of *S. enterica* serovars revealed sequence variations and belonged to sequence types II–VI. This study revealed that nucleotide substitution in the *invA* gene was more predominant between 40 bp and 250 bp positions. Further, it was observed that S. enterica strains isolated from chickens were represented in five sequence types (I, II, IV, V, and VI). Among these, types IV, V, and VI were represented by single strains with accession numbers *S. enterica* serovar Ruanda strain P13e (PP921854), *S. enterica* serovar Stockholm strain B5 (PP921857), and *S. enterica* serovar Chardan strain B7 (PP921858), respectively. Furthermore, two strains from free-ranging wildlife were observed to be unique in the sense that each belonged to a different sequence type.

This study further showed transition substitution along the sequence chains of *S. enterica* serovars at base pair positions C133T (*S. enterica* serovar Stockholm strain B5 [PP921857]), T220C (*S. enterica* serovar Heidelberg strain 670 [PP921861], *S. enterica* serovar Hadar strain B1 [PP921856], S. enterica serovar Stockholm strain B5 [PP921857], and S. enterica serovar Chardan strain B7 [PP921858]), C238T (*S. enterica* serovar Pomona strain SI-1 [PP921852]), and G250A (*S. enterica* serovar Heidelberg strain 670 [PP921861]). On the other hand, transversion substitution was identified in two *S. enterica* serovars at base pair positions C40G (*S. enterica* serovar Stockholm strain B5 [PP921857]), A210C (*S. enterica* serovar Ruanda strain P13e [PP921854]), and T211A (*S. enterica* serovar Ruanda strain P13e [PP921854]).

**Figure 2:**
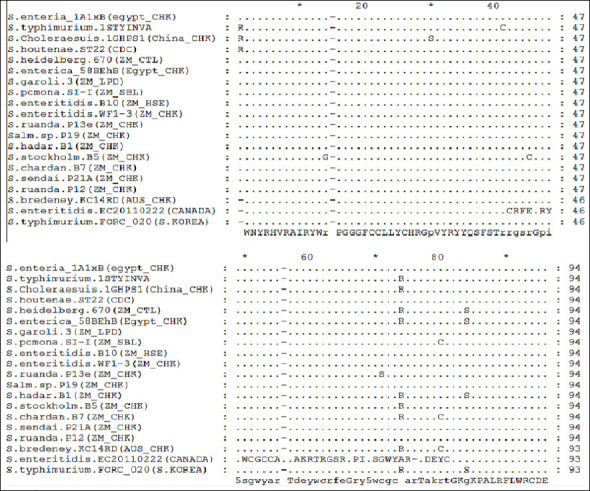
Multiple alignments of amino acid sequences of protein invasion A (*invA*-284 bp) sequences of local *Salmonella enterica* serovar strains isolated from domestic animals and wildlife compared with *S. enterica* strains published in GenBank.

The nucleotide substitution may result in significant protein structural changes in some strains and, in others, lead to silent protein structures (Figure 2 and Table 6). Interestingly, six sequences belonging to sequence type I with accession numbers *S. enterica* serovar Garoli strain 3 (PP921851), *S. enterica* serovar Enteritidis strain B10 (PP921862), *S. enterica* serovar Enteritidis strain WFI.3 (PP921853), *S. enterica* spp. strain P19 (PP921855), *S. enterica* serovar Sendai strain P21 (PP921859), and *S. enterica* serovar Ruanda strain P12 (PP921860) were not susceptible to substitution and hence maintained a similar amino acid sequence pattern arginine/proline/ arginine/arginine/cysteine/alanine/arginine/glutamine (RPRRCARQ) at positions 14, 30, 41, 45, 71, 74, and 84, respectively.

**Table 6:** Comparison of amino acid substitution in *invA*-284 bp gene sequences of local *Salmonella* *enterica* strains with GenBank reference strains.

S/N	Serotype/strain	Host	14	30	41	45	71	74	80	84
			—	R	P	R	R	C	A	R
1	*S.* enterica_1A1XB (Egypt)	Chicken	.	.	.	.	.	.	.	.
2	*S.* Typhimurium.1STYINVA	NK	.	.	C	.	.	R	.	.
3	*S.* Choleraesuis.1GHPS1	Chicken	.	S	.	.	.	R	.	.
4	*S.* Houtenae.ST22	Chicken	.	.	.	.	.	.	.	.
5	*S.* Heidelberg.670	Cattle	.	.	.	.	.	R	.	S
6	*S.* enterica_58BEhB	Chicken	.	.	.	.	.	R	.	S
7	*S.* Garoli.3	Leopard	.	.	.	.	.	.	.	.
8	*S.* Pomona.SI-I	Sable	.	.	.	.	.	.	C	.
9	*S.* Enteritidis.B10	Horse	.	.	.	.	.	.	.	.
10	*S.* Enteritidis.WF1-3	Chicken	.	.	.	.	.	.	.	.
11	*S.* Ruanda.P13e	Chicken	.	.	.	.	S	.	.	.
12	*Salmonella* spp. P19	Chicken	.	.	.	.	.	.	.	.
13	*S.* Hadar.B1	Chicken	.	.	.	.	.	R	.	S
14	*S.* Stockholm.B5	Chicken	G	.	.	C	.	R	.	.
15	*S.* Chardan.B7	Chicken	.	.	.	.	.	R	C	.
16	*S.* Sendai.P21	Chicken	.	.	.	.	.	.	.	.
17	*S.* Ruanda.P12	Chicken	.	.	.	.	.	.	.	.
18	*S.* Bredeney.KC14RD	Chicken	.	.	.	.	.	R	C	.
19	*S.* Enteritidis.EC20110222 (Canada)	Unknown	.	.	.	E	G	.	Y	.
20	*S.* Typhimurium.FORC_020 (S. Korea)	Unknown	.	.	.	.	.	R	.	S

*invA* = invasion protein A gene bp = base pairs S. = *Salmonella* spp. = species (plural) R = Arginine, P = Proline, C = Cysteine, A = Alanine, Q = Glutamine, S = Serine, G = Glycine, E = Glutamic acid, Y = Tyrosine. = no change (identical to consensus) NK = Not Known

Homology percentage of nucleotide sequences of the *invA* gene of *S. enterica* serovars revealed nucleotide similarities of 98%–100% among the 12 strains isolated from domestic animals and free-ranging wildlife (Table 7). While sequence comparison of the *invA* gene of local *S. enterica* strains with eight reference strains (*S. enterica* subsp. enterica [KJ718884.1], *S. enterica* subsp. enterica serovar Houtenae [DQ644627.1], *S. enterica* subsp. enterica serovar Choleraesuis [1GHPS1], *S. enterica* subsp. enterica serovar Typhimurium [M90846.1], *S. enterica* subsp. enterica [KJ718878.1], *S. enterica* serovar Typhimurium [CP012144.1], *S. enterica* subsp. enterica serovar Bredeney [KP279306.1], and *S. enterica* serovar Enteritidis [CP007323.1]) from GenBank showed 74.0%–100.0% identity.

**Table 7:** Homology percentage (%) of the nucleotide sequence of *invA*-284 bp gene in *Salmonella* *enterica* serovars.

No.	Isolate	Isolates from domestic animals and wildlife (n = 12)											
		5	7	8	9	10	11	12	13	14	15	16	17
5	*S.* Heidelberg	-	99	98	99	99	98	99	100	98	99	99	99
7	*S.* Garoli	1.0		99	100	100	99	100	99	98	99	100	100
8	*S.* Pomona.	2.0	1.0		99	99	98	99	98	98	99	99	99
9	*S.* Enteritidis.B10	1.0	0.0	1.0		100	99	100	99	98	99	100	100
10	*S.* Enteritidis.WF1	1.0	0.0	1.0	0.0		99	100	99	98	99	100	100
11	*S.* Ruanda.P13	2.0	1.0	0.0	1.0	1.0		99	98	98	98	99	99
12	*Salmonella* spp.	1.0	0.0	1.0	0.0	1.0	1.0		99	98	99	100	100
13	*S.* Hadar	0.0	1.0	0.0	1.0	0.0	0.0	1.0		98	99	99	99
14	*S.* Stockholm	2.0	1.0	1.0	1.0	2.0	1.0	1.0	1.0		98	98	98
15	*S.* Chardan	1.0	0.0	0.0	0.0	1.0	0.0	1.0	0.0	0.0		99	99
16	*S.* Sendai*.*	1.0	0.0	1.0	0.0	0.0	1.0	0.0	1.0	0.0	1.0		100
17	*S.* Ruanda.P12	1.0	0.0	1.0	0.0	0.0	1.0	0.0	1.0	0.0	1.0	1.0	

### Phylogenetic analysis of *invA* gene

**Figure 3:**
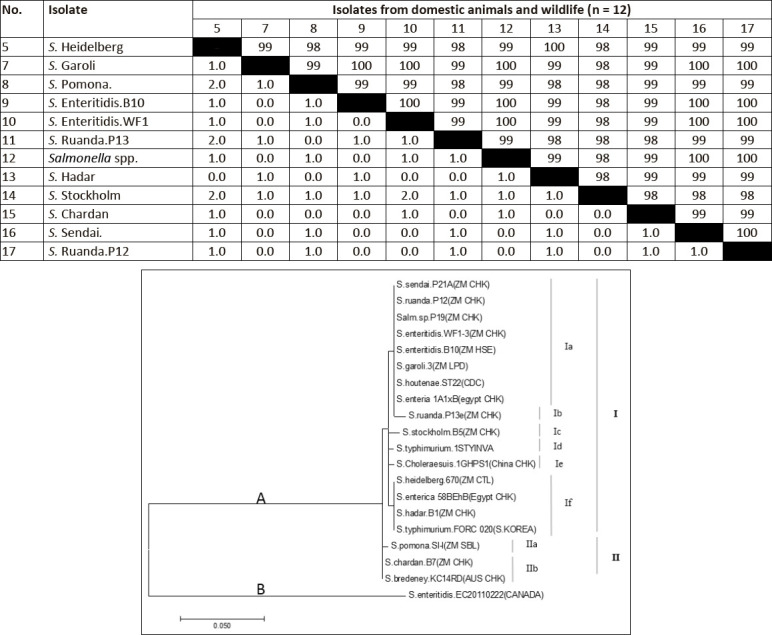
Phylogenetic analysis of protein invasion A (*invA*) gene nucleotide sequences of *Salmonella enterica* strains isolated from domestic animals and free-ranging wildlife by maximum-likelihood method based on the Tamura–Nei evolutionary model [20]. The distance scale was estimated at 0.050. The sequences under this study are marked Zambia (ZM).

To further amplify the relationship among *invA* sequences at the serotype level and source, a phylogenetic tree was constructed (Figure 3). The phylogenetic analysis of the *invA* gene sequences revealed two major lineages (Clade A and Clade B), with Clade A displaying a further eight subgroupings (a–h). All 12 sequences from domestic animals and free-ranging wildlife of Zambia belonged to Clade A (Figure 3). Clade B was represented by a single reference strain S. enterica serovar Enteritidis strain EC20110222 (CP007323.1) isolated from Canada and formed an out-group lineage.

## DISCUSSION

### Global significance of *S. enterica* and role of *invA* gene

*S. enterica* serotypes are a common cause of gastroenteritis and bacteremia infection worldwide [29–32]. The *invA* virulence gene is the most common and significant genetic marker in most, if not all, *S. enterica* subsp. enterica strains that cause salmonellosis globally [33]. In addition, the *invA* gene is an important marker in the identification of the *Salmonella* genus. Previous studies attempted to collate and compare *Salmonella invA* gene variation from a wide range of potential animal and human sources [13, 24].

### Conservation and hotspot regions of the *invA* gene

Similarly, the present study investigated the molecular diversity of the *invA* virulence gene in *S. enterica* strains isolated from domestic animals and free-ranging wildlife in selected areas of Zambia. In the present study, we observed that all serotypes were stable in the regions between positions 1–39 bp and 251–284 bp, suggesting that these regions are highly conserved within the *invA* 284 bp gene. This may underscore the significant role of the *invA* gene in ensuring that polymerase chain reaction-based diagnostic tests are broadly applicable across *S. enterica* serotypes [11–14]. However, the region between 40 bp and 250 bp in the nucleotide sequence of the *invA* 284 bp gene showed a likelihood of being a hotspot.

### Genetic variability and evolutionary implications

In this region, substitutions of single-nucleotide polymorphisms were observed, resulting in distinct sequence types while maintaining the unique nucleotide sequence targeted to identify bacteria of the *Salmonella* genus. These hotspots are genomic regions characterized by high rates of recombination, mutation, or integration of foreign DNA [34, 35]. Within *Salmonella* pathogenicity island 1, this region facilitates lateral gene transfer and adaptive evolution by promoting the integration of foreign genetic elements such as plasmids, transposons, or bacteriophages. Therefore, the detection of multiple *invA* sequence types, including unique variants not previously reported in GenBank, underscores the dynamic evolution of *S. enterica* in mixed-animal ecosystems.

These findings expand the current understanding of *invA* gene plasticity and provide a genetic basis for refining molecular surveillance and diagnostic assays in sub-Saharan Africa. For instance, the polymorphic regions identified in the *invA* nucleotide sequences of domestic and wild animal isolates could help design Zambia-adapted PCR primers, reducing the likelihood of false-negative detections due to sequence variability. The submission of GenBank sequences PP921851–PP921862 adds valuable regional reference data for global *Salmonella* databases and bioinformatics benchmarking.

### Host-associated diversity and antibiotic selection pressure

Further, this study reports *invA* sequence variations among intra- and inter-serovar *S. enterica* strains, although partial sequencing of the full-length 2176 bp *invA* gene was performed. Interestingly, sequences of *S. enterica* strains from chickens were more diverse. This can be attributed to sampling bias and the discriminatory use of antibiotics in the poultry industry, as well as among backyard poultry keepers, which may select for resistant *S. enterica* strains and subsequently impact the *invA* gene [36].

### Functional implications of amino acid substitutions

Furthermore, the study demonstrated that nucleotide substitutions in this region resulted in significant amino acid changes in some strains from domestic animals and free-ranging wildlife. For instance, nucleotide substitution in *S. enterica* serovar Heidelberg strain 670 (PP921861) at position 220 bp resulted in amino acid changes from alanine-to-arginine at position 74 (Figure 2 and Table 6). This phenomenon was observed in at least 45% (9/20) of sequences from both local and reference strains, suggesting that alanine is likely to be substituted by arginine at position 74. The findings of this study indicate that substitution of alanine with arginine at amino acid position 74 in the *invA* gene may be associated with structural alterations in the protein, potentially affecting the functionality of the type III secretion system or contributing to host adaptation [37], particularly if the residue resides within a transmembrane helix or a tightly packed structural domain.

Topology analysis, secondary structure prediction, and mutation stability assessments were not conducted because they were beyond the scope of the present study. The *invA* gene encodes invasion protein A, a key component of the *Salmonella* type III secretion system, which facilitates host cell invasion and colonization [15]. Therefore, substitution of alanine by arginine in the variable region of the *invA* gene could influence traits such as the ability to evade host innate immune responses or adapt to different host niches [38–40]. However, whether the genetic variations observed in some *S. enterica* strains in the present study indicate increased or reduced virulence remains unknown. Functional studies are therefore required to determine whether these variations affect virulence, host specificity, or environmental persistence.

### Comparative genomic insights

In contrast, the present study shows that glutamine substitution at position 84 leads to silent substitution changes (serine). In Egypt, a similar study showed multiple nucleotide substitutions in the sequence chain of *S. enterica* serovar Typhimurium strains between positions 208 bp and 1813 bp of the 2058 bp *invA* gene [13]. That study revealed amino acid substitution from serine to phenylalanine at residue number 530. From these studies, it may be noted that pathogenic bacteria can evolve over time to develop strategies that facilitate survival or propagation within a host, causing infection and spreading disease [41, 42].

Factors associated with rapid generation rates of substitution in amino acid sequences may include selection for antigenic diversity to escape host immune responses [43]. Low or no nucleotide substitution was observed in some *S. enterica* strains from Zambia, such as *S. enterica* serovar Enteritidis strain B10 (PP921862), S. *enterica* serovar Sendai (PP921859), *S. enterica* serovar Garoli strain 3 (PP921851), *S. enterica* spp. (PP921855), and *S. enterica* serovar Ruanda (PP921860), despite differences in serotype and host source.

### Zoonotic transmission and ecological interface

The findings of the present study demonstrate a close genetic relationship between isolates originating from domestic and wildlife hosts, suggesting the presence of potential bidirectional zoonotic transmission pathways within Zambia’s ecosystem. The nucleotide diversity of *S. enterica* isolates from clinically symptomatic chickens in Zambia (74%–100%) aligns closely with reports from clinically symptomatic chickens in China (72.9%–97.6%) [44] but is notably lower than that observed in poultry samples from Egypt (99.2%–99.4%) [13].

In the same study, a higher homology percentage similarity (99.2%–99.4%) for nucleotide sequences and (99.6%–99.9%) for amino acid sequences were recorded between Egyptian local isolates and other published sequences in GenBank [13]. These comparisons highlight both regional variations and unique evolutionary patterns of *S. enterica* lineages across different ecological and geographic contexts.

In the present study, the international reference *S. enterica* serovar Enteritidis strain (CP007323.1) from Canada showed more nucleotide substitutions than local *S. enterica* serovar Enteritidis strains from horse (PP921862) and chicken (PP921853). All local *invA* nucleotide sequences obtained from domestic and wildlife isolates showed ≤98% similarity to one or more known international GenBank reference sequences and were considered putative novel *invA* allelic variants.

### Implications for diagnostics and surveillance

The detection of putative novel *invA* allelic variants underscores the dynamic evolution of *S. enterica* within diverse ecological and host settings. Such genetic changes may influence virulence and transmission potential while undermining the sensitivity of *invA*-based diagnostic assays widely used in surveillance programs. Sustained genomic monitoring and routine validation of diagnostic tools are therefore critical to ensure early detection, accurate outbreak tracing, and effective public health response to emerging *Salmonella* strains.

Further, the study observed that all Zambian isolates clustered within Clade A, indicating localized lineage evolution potentially influenced by antibiotic selection pressures or ecological interactions between domestic animals and wildlife. Three sequences from domestic animals (*S. enterica* serovar Ruanda strain P13e [PP921854], *S. enterica* serovar Stockholm strain B5 [PP921857]) and wildlife (*S. enterica* serovar Pomona [PP921852]) each represented distinct groups, suggesting that they were genetically not closely related to either local or reference strains.

### Ecological adaptation and One Health perspective

Bacteria can adapt to various adverse environmental conditions, thereby facilitating their survival. Adaptations enable bacteria to overcome challenges and evolve as successful pathogens, potentially resulting in severe disease outcomes [38, 45]. This study observed sequence diversity in the *invA* gene across hosts, highlighting shared and divergent genomic signatures that may underpin cross-species transmission dynamics.

Therefore, understanding these molecular variations provides critical insight into how ecological overlap and pathogen exchange at the wildlife–livestock interface contribute to the broader zoonotic *Salmonella* reservoir, informing integrated surveillance, biosecurity, and public health interventions under the One Health framework. To the best of our knowledge, this is the first report from Zambia demonstrating nucleotide sequence variation in the *invA* gene of *S. enterica* strains isolated from both domestic and wildlife hosts within a single molecular dataset using short-target phylogenetic inference based on a 284 bp *invA* gene fragment.

Our findings show that even a short gene fragment can provide substantial phylogenetic resolution, enabling inference of relationships among isolates and comparison of nucleotide diversity with global reference strains. Therefore, the present study provides a foundational dataset to support regional molecular surveillance through full genome sequencing of *Salmonella* virulence genes in southern Africa, bridging wildlife ecology and livestock epidemiology.

### Limitations and future perspectives

This study has several limitations. First, only a fragment of the *invA* gene was sequenced, which may under-represent total nucleotide variation and limits phylogenetic resolution and detection of variants outside the amplified region. Second, the number of wildlife-derived *S. enterica* isolates was small, and sampling was geographically and temporally constrained, reducing statistical power and generalizability. Third, primer choice and amplification in the PCR may have introduced bias against divergent alleles.

Additionally, comprehensive phenotypic and epidemiologic metadata linking sequence variation to virulence or resistance were lacking. Although standard laboratory controls were applied, sequencing or amplification artifacts cannot be completely excluded. These limitations highlight the need for expanded sampling and validation through full-gene or whole-genome sequencing with paired phenotypic characterization in future studies.

Future studies should include whole *invA* or whole-genome sequencing, increase wildlife sample diversity, include multiple seasons and locations, and integrate genetic data with epidemiologic metadata.

## CONCLUSION

This study demonstrated distinct nucleotide sequence variations in the *invA* gene among *S. enterica* isolates from domestic animals and free-ranging wildlife in Zambia. Despite the overall high nucleotide similarity (98%–100%) among local isolates, six distinct sequence types were identified, with substitutions predominantly occurring between positions 40 bp and 250 bp. Phylogenetic analysis revealed that all isolates clustered within a single lineage (Clade A), indicating close genetic relatedness across host species. Notably, certain nucleotide substitutions resulted in amino acid changes, particularly alanine-to-arginine at position 74, suggesting potential functional implications for the invasion protein.

These findings have important practical implications for molecular diagnostics and surveillance. The identification of polymorphic regions within the *invA* gene highlights the possibility of reduced sensitivity of standard PCR assays when applied to genetically diverse regional strains. Therefore, region-specific primer design and continuous validation of diagnostic tools are essential to improve detection accuracy. Furthermore, the observed genetic similarity between isolates from domestic animals and wildlife underscores the potential for bidirectional zoonotic transmission, emphasizing the need for integrated surveillance strategies under the One Health framework.

A major strength of this study is the inclusion of *S. enterica* isolates from both domestic animals and wildlife within a single analytical framework, allowing direct comparison of genetic diversity across ecological interfaces. In addition, the use of sequence-based analysis provided deeper insights beyond conventional detection methods, enabling the identification of subtle genetic variations and phylogenetic relationships. The integration of local isolates with global reference sequences further enhanced the robustness of comparative analysis.

However, several limitations should be considered. The study focused on a partial fragment of the *invA* gene (284 bp), which may not fully capture the complete extent of genetic variation. The relatively small number of wildlife-derived isolates and restricted sampling locations may limit the generalizability of the findings. Additionally, the absence of phenotypic and epidemiological data restricts the ability to correlate genetic variation with virulence, antimicrobial resistance, or transmission dynamics.

Future studies should employ full-length *invA* gene sequencing or whole-genome sequencing to achieve higher-resolution assessment of genetic diversity. Expanding the number and diversity of wildlife and livestock samples across multiple regions and seasons will improve representativeness. Integrating genomic data with phenotypic, ecological, and epidemiological information will be critical for understanding the functional significance of observed mutations and their role in host adaptation and disease transmission.

In conclusion, this study provides novel insights into the molecular diversity of the *invA* gene in *S. enterica* at the wildlife–livestock interface in Zambia. The findings highlight ongoing genetic evolution, potential zoonotic transmission pathways, and the need for continuous genomic surveillance to support effective disease control and public health interventions.

## DATA AVAILABILITY

The supplementary data are available from the corresponding author upon reasonable request.

## AUTHORS’ CONTRIBUTIONS

CMM, BHM, and JBM: Conceived and designed the study. CMM and JN: Performed the study. ES, JN, and CMM: Analyzed and interpreted the data. CMM: Drafted and revised the manuscript. All authors have read and approved the final version of the manuscript.
